# Mechanistic Roles of Transcriptional Cyclin-Dependent Kinases in Oncogenesis: Implications for Cancer Therapy

**DOI:** 10.3390/cancers17091554

**Published:** 2025-05-03

**Authors:** Mohammed Alrouji, Mohammed S. Alshammari, Saleha Anwar, Kumar Venkatesan, Anas Shamsi

**Affiliations:** 1 Department of Medical Laboratories, College of Applied Medical Sciences, Shaqra University, Shaqra 11961, Saudi Arabia; malrouji@su.edu.sa; 2 Department of Clinical Laboratory Sciences, College of Applied Medical Sciences, Shaqra University, Shaqra 11961, Saudi Arabia; m.alshammari@su.edu.sa; 3 Center for Interdisciplinary Research in Basic Sciences, Jamia Millia Islamia, New Delhi 110025, India; email2saleha@gmail.com; 4 Department of Pharmaceutical Chemistry, College of Pharmacy, King Khalid University, Abha 62529, Saudi Arabia; kumarve@kku.edu.sa; 5 Centre of Medical and Bio-Allied Health Sciences Research, Ajman University, Ajman P.O. Box 346, Saudi Arabia

**Keywords:** cyclin-dependent kinases (CDKs), transcriptional CDKs, CDK inhibitors, cancer therapeutics, kinase-targeted therapy

## Abstract

Cyclin-dependent kinases (CDKs) are pivotal in regulating cell cycle progression and transcription, making them crucial targets in cancer research. The two types of CDKs that regulate different biological activities are transcription-associated CDKs (e.g., CDK7, 8, 9, 12, and 13) and cell cycle-associated CDKs (e.g., CDK1, 2, 4, and 6). One characteristic of cancer is the dysregulation of CDK activity, which results in unchecked cell division and tumor expansion. Targeting transcriptional CDKs, which control RNA polymerase II activity and gene expression essential for cancer cell survival, has shown promise as a therapeutic approach in recent research. While research into selective inhibitors for transcriptional CDKs is ongoing, inhibitors that target CDK4/6, such as palbociclib and ribociclib, have demonstrated encouraging outcomes in treating breast cancer. CDK7, CDK8, and CDK9 are desirable targets for therapy since they have shown oncogenic roles in a variety of cancer types, such as colorectal, ovarian, and breast malignancies. Even with significant advancements, creating selective inhibitors with negligible off-target effects is still difficult. This review highlights the need for more research to optimize therapeutic strategies and improve patient outcomes by giving a thorough overview of the non-transcriptional roles of CDKs in cancer biology, their therapeutic potential, and the difficulties in targeting these kinases for cancer treatment.

## Simple Summary

One of the primary features of cancer is unchecked cell growth, which is frequently brought on by proteins known as cyclin-dependent kinases that control when and how cells divide or manufacture vital molecules like RNA. Tumor growth may result from the improper function of these proteins. Patients with certain tumors, such as breast cancer, are benefitting from medications that already target these particular kinds of proteins. It is more difficult to safely and successfully target other kinds of these kinases, particularly those that are involved in gene expression. This article examines the role of these lesser-known kinases in cancer, the advancements made in developing novel medications that target them, and the obstacles still facing the development of more effective therapies. The goal is to help researchers find more effective ways to stop cancer cells while minimizing harm to healthy cells.

## 1. Introduction

Cell division and cell death are the major predominant metabolic processes in maintaining tissue homeostasis in mammals. The cell-cycle process has resisted changes over evolutionary time and hence is a highly conserved process. The precisely conserved process governs cell division and consists of the following phases: i. the G0/G1 phase or the gap phase, ii. the synthesis phase, S phase, and iii. the Gap2 (G2) and Mitosis (M) phase. Multiple checkpoints ensure the regulation of replication in the S phase [1,2]. G1 and G2 phases act as major checkpoints between the G1 and S phases and govern the fate of the cells. The cell may enter the S phase or halt at the G0 phase by exiting the cell cycle under the regulation of many cyclins and their association partner CDKs. CDKs make complexes with their specific cyclins for their activation and stabilization [2]. CDKs without their corresponding cyclin are unable to generate any functional response, and the CDKs alone are 40 thousand times less active in comparison to the non-covalent dimer complex [3,4]. The cyclin-CDK regulates the cell cycle progression and transcription. It governs the cell cycle by phosphorylating target genes such as retinoblastoma (Rb). Mitogenic signals are responsible for activating the CDK-cyclin complex, and the complex is subdued in response to any DNA damage at the cell-cycle checkpoints [5]. Through their highly conserved PSTAIRE motif, CDKs bind with cyclins to produce a dimer that is shared by all isoforms [6]. The interaction leads to a series of changes in the conformation of the protein kinase associated with its activity. The T-loop of the protein kinase undergoes a conformational reorientation of the L12 helix into beta-strand, altering the function of the active site and T-loop [7]. The enzymatic activity of CDKs suffers a huge loss when dissociated with its activity partner, cyclin; the loss of activity is probably due to modulation in the kinase structure that hinders the active site [8]. The CDKs are subjected to ubiquitinization-mediated inactivation after the completion of their activity. Apart from cyclins, CDK activity is governed by various other phosphatases and kinases, known as CDK-activating kinase, which acts as a positive regulator of the kinase and adds phosphates to threonine residue in the active site [9].

**Figure 1:**
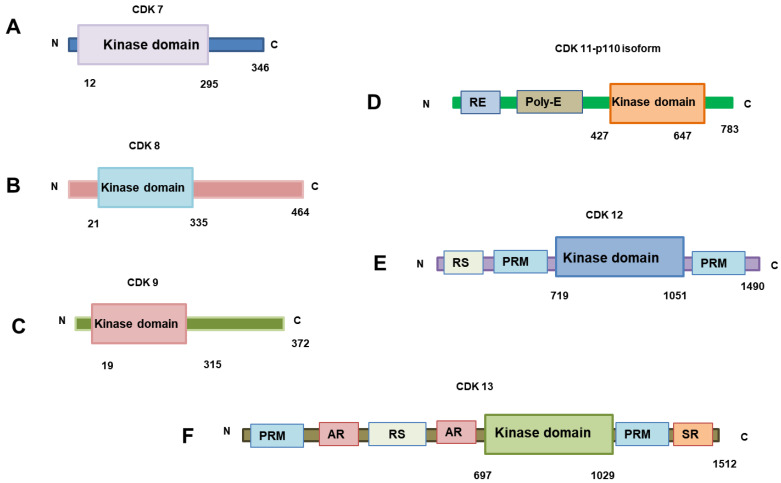
The figure highlights the structure of the transcriptional CDKs and their important domains. (**A**) CDK7; (**B**) CDK8; (**C**) CDK9; (**D**) CDK11; (**E**) CDK12; (**F**) CDK13. (RS domain = Arginine-Serine-rich domain; PRM = Proline-Rich Motif; AR domain = Alanine-Rich domain; SR domain = Serine-Arginine-rich domain).

Signal transduction pathways depend heavily on protein kinases, and many disorders have been linked to abnormal kinase activity. Kinase inhibition has emerged as a key therapeutic intervention area in recent years, and several kinase inhibitor pharmacophores have been approved [10] as regions where ATP binds to give phosphate groups. Most CDK inhibitors, such as THZ1, Abemaciclib, and Palbociclib, are ATP-competitive, which means that they occupy this site and prevent substrate phosphorylation. Figure 1 shows all the transcriptional CDKs, highlighting their kinase domain and structural features.

Aberrant progressions in cell cycles are considered one of the major mechanisms contributing towards cancer [11]. In its un-phosphorylated form, Rb protein prevents G1 to S phase transitions by interacting with E2F transcription members. The associations prevent E2F activation as well as repress transcription by attaching the deacetylases to the promoter of S phase entry genes [12]. Rb is incapacitated in the cell cycle by a sequence of events involving the CDKs 4 and 6 altogether with cyclin E and cdk2, forming a complex [13,14]. The mitotic events further lead to the synthesis of D-type cyclins that are associated with CDK 4 and CDK6. The process requires involvement from the Cip/Kip family members, which are potent inhibitors of CDK2 and also work to activate dependent kinases (CyclinD-DKs) [15]. CyclinD-DKs are associated in a series of events associated with G1 progression, S phase entry, and progression of the cell cycle. Cyclin D-DKs phosphorylate Rb, releasing the repressed transcriptional machinery by the RB-E2F complex. It further quenches the Cip/Kip proteins to activate CDK2 and its activating partner Cyclin E, which is associated with adding phosphate groups to Rb, disrupting the Rb-E2F complex formation. The overall events allow the activation of E2F and transcription of genes associated with entry to the synthesis phase [16].

**Figure 2:**
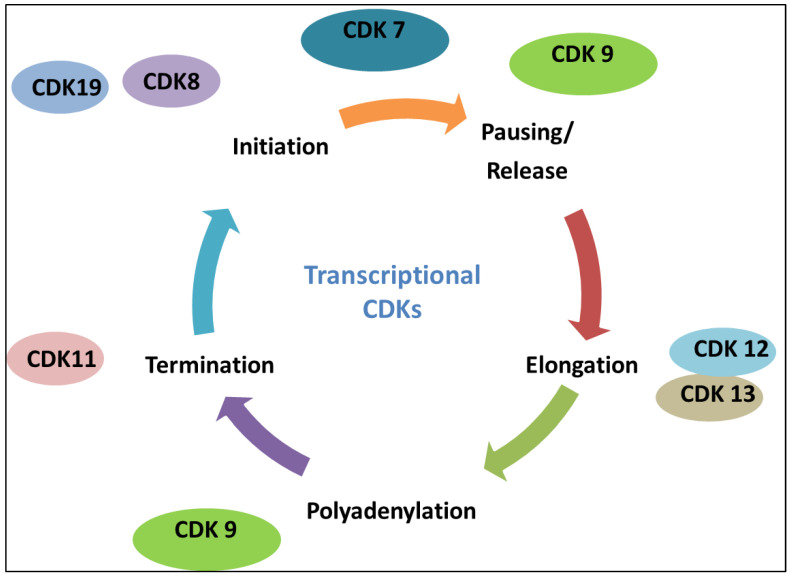
The roles of transcriptional CDKs in regulating gene expression are shown in this diagram. Transcription start is aided by CDK7, polyadenylation and pausing/release are controlled by CDK9, CDK12/CDK13 maintains elongation, and termination is managed by CDK11. The sequential and coordinated activity of CDKs across transcriptional stages is highlighted by the modulatory role of CDK8/CDK19 in initiation.

Cancer is a complex disease and includes many genetics and environmental factors that endorse its incidence. Unfortunately, cancer pervasiveness has been growing in such a way that it is considered the subsequent chief cause of death after heart-related ailments globally [17]. As per the reports by the American International Cancer Agency, one in five is prone to develop cancer in their lifetime. Cancer prevention has emerged as the most challenging milestone in public health development (https://www.iarc.who.int/cancer-topics/, accessed on 6 March 2025). One of the major hallmarks of cancer is altered metabolism, contributing to acquiring more nutrition and abnormal proliferation of cells [18]. CDKs, as the regulators of the cell cycle, also regulate cell proliferation in mammals [19,20]. An imbalance in the cell cycle machinery caused by the over-activation of CDKs results in unchecked cell division, which improves the prognosis of malignant tumors [21]. As a result, the cell cycle machinery regulators are regarded as important cancer treatment targets. Numerous pieces of evidence suggest that the cell cycle regulation system contributes to various cancer hallmarks. Therefore, treatments that target the elements of the cell cycle machinery can help stop the growth of cancer cells [22]. Over 20 serine/threonine kinases that regulate fundamental cellular functions comprise the cyclin-dependent kinase (CDK) family. The CDKs are engaged in many distinct cellular processes and can be broadly divided into two groups: (i) CDKs associated with phases of the cell cycle (CDK1, 2, 4, and 6) and (ii) CDKs associated with transcription (CDK7, 8, 9, 12, and 13) as shown in Figure 2 [23]. Transcription-associated CDKs (tCDKs) control gene transcription by phosphorylating the DNA-directed RNA polymerase II subunit (RPB1) of RNA polymerase II (RNA Pol II) at its C-terminal domain (CTD) [24,25]. Figure 2 shows the role of transcriptional CDKs at different phases of transcription.

CDKs govern the processes involved in cancer survival and progression [26,27,28] and therefore have been targeted as promising targets in oncology [29]. Inhibitors of CDK4/6, such as palbociclib and ribociclib, gained wide recognition in recent years in hormone receptor-positive breast cancer. They show remarkable improvements in the overall survival of breast cancer patients [30,31]. Cell cycle-targeting inhibitors are presently undergoing clinical studies for various cancers, whereas tCDKs are less developed as cancer therapeutic targets and have not yet seen the introduction of small-molecule inhibitors into clinical trials.

## 2. CDKs: Roles in Cell Cycle

In addition to having diploid DNA, most adult cells are in a quiescent state (G0), which can be either temporary or permanent. In the mitotic phase, quiescent cells re-enter the cell cycle when triggered by mitogens such as growth factors. CDK4/6 is activated by the external stimuli, which starts a series of intracellular signals moving the cell cycle from the quiescent G0/G1 phase to the synthesizing S phase. The activity of CDK4/6 is controlled by its contacts with D-cyclins and inhibited by its interactions with the INK family’s CDK inhibitors (CDKIs) [32,33,34]. Recent studies have clarified a number of previously unknown roles for CDK6 and point to a new function for CDK6 as a transcriptional regulator that connects differentiation and other cellular processes to cell-cycle progression. Several tumor suppressor proteins, including protein RB, p107, and p130, are phosphorylated and inhibited by the complex that results from CDK4/6’s interaction with cyclin D. The RB protein slows the G1-S transition by blocking the E2F transcription factor (E2FTF), which controls genes via enlisting co-repressors. Thus, the cell cycle advances from the G1 phase to the synthesis phase when RB is inactivated by a phosphorylation cascade. Meanwhile, phosphorylated RB represses E2F once more, which triggers the transcription of G1-S target genes such as dihydrofolate reductase (DHFR), cyclin E, polo-like kinase 1, and others that are crucial for cell cycle progression through G1-S phase entry [35]. Target genes, including cyclins E1 and E2, are activated in late G1, and, through their interactions with CDK2, they further activate CDK2. CDKIs p21CIP1 and p27KIP seize CDK2, and the kinase is vulnerable to ubiquitin-mediated destruction [36].

Moreover, CDK2, in its active form, can phosphorylate various proteins required in various important cell events such as the progression of the cell cycle, factors for DNA repair, the duplication of centrosome, and the synthesis of histone [37,38]. Specifically, after the S phase begins, the cyclin E/CDK2 complex regulates RB to replace the G1/S phase’s restriction point at the edge, creating a positive feedback loop. At the end of the S phase, cyclin A removes cyclin E, and a new cyclinA-CDK2 complex is created, in which cyclin E rapidly degrades via ubiquitylation [39,40]. In order to end the S phase and prevent it from continuing into the G2 phase, the CDK2 complex with cyclin A now phosphorylates CDC6 and E2F1. Cyclin A then helps cells reach the M phase of the cell cycle by activating CDK1. Chromosome condensation, mitotic spindle construction, and nuclear envelope rupture are all caused by phosphorylated CDK1. In the M-phase of the cell cycle, the spindle assembly checkpoints regulate the cell transitions from the metaphase to the anaphase. The start of the anaphase is caused by APC/C’s degradation of the CDK1-cyclin B complex [41]. Further, after CDK1 deregulation, the chromosomes are separated, and mitosis comes to an end. The chromosomes are separated, and mitosis comes to an end, followed by cytokinesis. CDK1 plays a significant role in cell-cycle progressions as it initiates the mitosis and ensures the replication with high fidelity [42].

**Figure 3:**
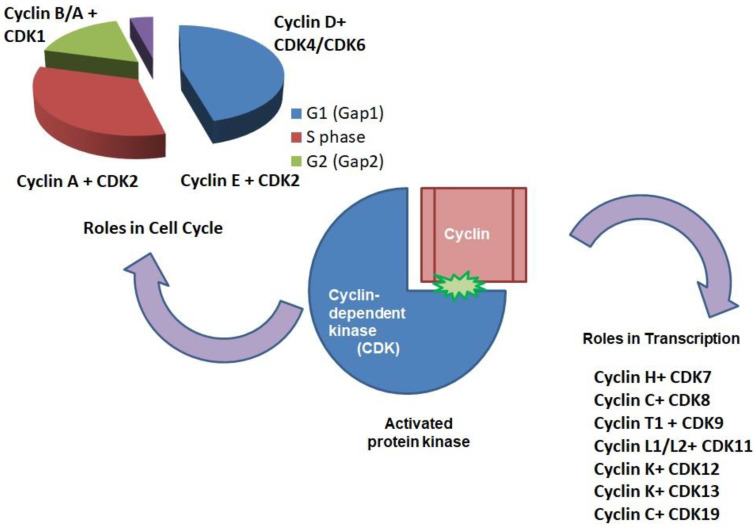
This figure illustrates the involvement of specific cyclin-CDK complexes in distinct phases of the cell cycle: Cyclin D with CDK4/6 in the G1 phase, Cyclin E with CDK2 in the G1/S transition, Cyclin A with CDK2 in the S phase, and Cyclin A/B with CDK1 in G2 and mitosis.

Figure 3 shows the interaction of CDK and cyclins at various phases of the cell cycle.

CDK1 associates with cyclin B1 and assists in the transition from the G2-M phase [43]. The protein kinase is regulated by other kinases such as checkpoint kinase 1 (CHK1) and Wee-1-like protein kinase (WEE1). The checkpoint kinases ensure omitting the damaged and incompletely replicated DNA from incorporation in the daughter cells [44]. CDK1, coupled with its partner cycB1, shows increased activity in the late G2 phase, and the enhanced activity continues throughout pro-metaphase till metaphase [42,45]. While CDK2 is associated with G1-S and S-G2 transitions, it also phosphorylates various important transcription factors, such as Myb-related protein B, Myc proto-oncogene, FOXO, nuclear factor-Y, etc. [46,47,48,49], which work together for transitions in different cell cycle phases. Additionally, the kinase regulates cell differentiation, proliferation, immune responses, and apoptosis; however, its overexpression is associated with cancer [50,51,52].

### Cyclin-Dependant Kinase 5

One of the most functionally varied kinases in neuronal cells is cyclin-dependent kinase 5 (Cdk5), a proline-dependent protein kinase (PDPK). Cdk5 works by attaching itself to its p35/p39 regulatory neuron-specific subunits. Cdk5–p35 regulates cellular and developmental processes in the brain, such as microtubule control and cell cycle modulations, depending on binding and function [53,54,55,56]. Cdk5 is a neural-specific kinase active in post-mitotic neurons, and only the nervous system can express its activators [57]. The pathophysiology of several neurodegenerative illnesses, including AD, is linked to the excessive activation of Cdk5 by the shortened p35 byproduct, p25 [58]. In addition to being expressed in the nervous system, CDK5 is essential for angiogenesis, gene expression, cell division, and differentiation [59,60]. CDK5 has an essential role in maintaining the blood glucose level by modulating the activity of pancreatic cells [61]. Although CDK5 is a major contributor to neurogenesis by aiding the embryonic development of the central nervous system (CNS), its aberrant expression results in disturbed synaptic homeostasis. CDK5 signaling aids in the reversal of damage under stressful conditions; however, prolonged activation of the CDK-p25 complex is closely associated with neuronal damage and cell death [62].

## 3. The Roles of CDKs in Transcription

**Figure 4:**
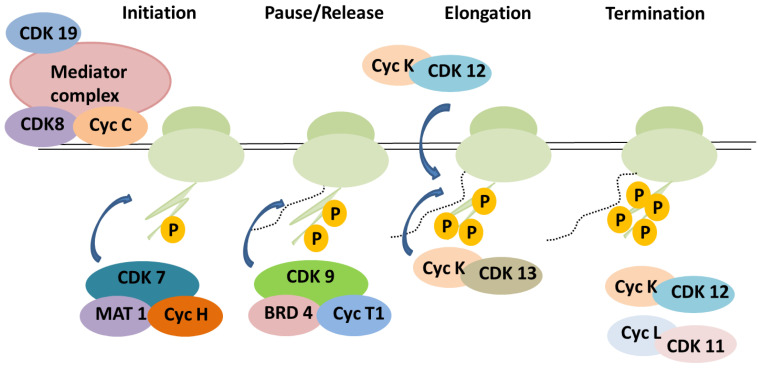
This figure illustrates transcription regulation stages, highlighting CDK-cyclin complexes. The pre-initiation, initiation, elongation, and termination phases of the RNAPII-based transcription cycle are controlled by different CDK/cyclin complexes at each stage.

In order to activate and further regulate gene transcription, transcriptional-associated CDKs, likely cell cycle CDKs, need a cyclin partner [6]. The 346 amino acid protein kinase CDK7/CDKN7 functions as a cyclin-activating kinase (CAK) in conjunction with its binding partner cyclin H and auxiliary protein MAT1. S164, an additional phosphorylation site inside the T-loop, distinguishes CDK7 from other CDKs. The site, when phosphorylated, facilitates CDK7 to aid in transcription; however, the phosphorylation is not associated with cell cycle regulatory mechanisms [63,64]. The kinase exerts its role in transcription by adding phosphate groups to the C-terminal domain (CTD) of RNA Pol II; it also regulates transcription initiation and promoter escape [65,66]. To activate CDK9, the CDK7 complex with its activating partners binds to the main TFIIH complex. In the T-loop, it phosphorylates CDK9′s Thr186. The transcription RNA Pol II is switched from the initiation phase to the elongation phase by the activation cascades [67]. CDK7 acts as a bridge for cells to move from the G1-S phase by phosphorylating the CDK2/cyclin E complex. After completion of the synthesis phase, CDK7 aids in the activation of the CDK1/cyclin-B complex, which acts as an important factor in the mitotic entry. Cyclin H is the activation partner for CDK7, and the association requires the phosphorylation of Thr170, located in the activation loop of CDK7 [63,68]. The role of transcriptional kinases is illustrated in Figure 4.

CDK8 is a 372 aa, Ser/Thr kinase, and, along with its paralog kinase, CDK19 is associated with a mediator complex in the cells [69]. CDK8 has many phosphorylation targets, such as the RNA polymerase II, histone H3, and various general transcription factors [70]. CDK9 binds to its cyclin partners, cyclin T/K, for its activation [71,72]. CDK9 is thought to play functions in RNA processing and replication-related stress responses. It preferentially confines to the non-nucleolar nucleoplasm with notable enhancement near nuclear speckles [73,74]. CDK8, when associated with the multimeric transcriptional mediator complex, generated signals to RNA Pol II [75]. The CDK8 complex also phosphorylated CDK7, inhibiting the activity of TFIIH to initiate transcription and its CTD activity. CDK19, a paralog of CDK8, also shows associations with cyclin C [2]. CDK19 can bind to the mediator complex to modulate transcriptional events and perform roles in p53 regulation [25]. CDK8 and 19 can phosphorylate as many as 60 other protein targets associated with transcription [76].

CDK12 was initially known as a CDC2-related kinase (CRKRS) with Arg/Ser-rich domains. CDK12 is a 1490 aa protein, which is one of the largest in the CDK family, and it consists of a CTD with a kinase domain, two proline-rich motifs that mediate interactions with other proteins, and an Arg/Ser (RS) domain common in splicing factors [77]. CDK12 plays a major role in embryo development. Implantations of CDK12 knockout genes containing blastocysts in mice lead to lethal conditions and fail to grow, leading to apoptosis and DNA damage [78]. L1 and L2 cyclins were identified as binding partners for CDK12; however, it was later established that cyclin K is the actual associating partner for CDK12 activation [79,80,81]. The CDK most comparable to CDK12 is CDK13, a protein that is 1512 aa long and has up to 50% sequence homology with CDK12. In contrast to CDK12, CDK13 has two alanine-rich domains and a C-terminal SR domain. Nonetheless, the two kinases are 92% identical inside their respective kinase domains. Structural studies of both kinases have demonstrated that the N-terminal lobe of the kinase domain of CDK12 borders with cyclin K at its cyclin box. This results in a CDK12-cyclin K heterodimer, similar to how CDK13 and cyclin K interact [81,82]. Although both kinases share structural similarities, they are very diverse in the regulation of genes [83]. Both the kinases work in shared yet non-overlapping functions [84]. Studies have revealed that CDK10 and 11 can enhance transcription. The association of the CDK11-cyclin L complex with various elongation factors (EFs) is associated with transcriptional elongation [85]. CDK11 is also associated with the progression of the cell cycle as it delays cell doubling time and telophase, and its knockdown results in mitotic arrest [86,87].

## 4. CDKs as Therapeutic Targets in Cancer Therapeutics

mRNA production is a highly sophisticated event involving strict and regulated control of all the involved events, from transcription to its export. Various proteins are associated with the formation of these complexes, such as interactions with DNA and pre-mRNAs. Within the transcriptional CDK sub-branch, CDK7, CDK8/19, and CDK9 are linked to TFIIH, the mediator complex kinase module (MED), and P-TEFb, respectively. CDK7 and CDK8 control transcription initiation, while CDK9 facilitates promoter-proximal release. CDK12 and CDK13 promote transcript synthesis at the mid and 3′-end of RNA and phosphorylate the CTD. Furthermore, CDK12 controls alternative last exon splicing and the expression of genes that react to heat shock, stress, and DNA damage [82,88,89,90,91]. The CDK10 complex targets substrates such as the protein kinase PKN2 and the ETS2 oncoprotein to control gene transcription independently of RNAPII phosphorylation. The CDK11 complex affects apoptosis, neuronal function, the cell cycle, RNA transcription, and splicing. The emergence of transcription-associated CDKs as therapeutic targets can be attributed to “transcriptional addiction”. Super-enhancers (SEs), specialized enhancers that drive high gene expression linked to cancer progression, are highlighted in recent data as being highly relied upon by cancer cells. By targeting transcriptional CDKs, SEs may be disrupted, affecting the transcription of oncogenic genes in cancer cells while preserving normal cells, opening up a new treatment option for cancer [92,93,94]. The sequential recruitment of enzymatic and regulatory co-factors is necessary for the transcription of particular genes. For example, CDK-containing initiation/elongation complexes, BRD4, and MED1 are involved in super-enhancer-regulated transcription. Complex tumors such as adult T-cell leukemia (ATL), pancreatic ductal adenocarcinoma (PDAC), TNBC, ovarian, and medullary thyroid cancers respond especially well to this approach. Pharmaceutical companies have developed pan-CDK and selective CDK inhibitors as promising cancer therapeutics because targeting the CDK family generally yields selective antitumor effects [95,96]. Table 1 shows the role of transcriptional CDKs in cancer.

## 5. CDK7 as a Therapeutic Target in Cancer

The kinase CDK7 is a significant constituent of the multiprotein complex CDK activating kinase (CAK) and a subunit of the multiprotein basal TF, TFIIH. One of the cell cycle’s master regulators, CDK7, phosphorylates another cell cycle CDKs. As a TFIIH subunit, the kinase phosphorylates the RPB1 subunit of RNA polymerase II to regulate the start of transcription. Serines 5 and 7 of RPB1’s hepta-peptide repeat exhibit phosphorylation in the C-terminal domain (CTD) [120]. Proliferating cells depend on DNA repair, cell cycle regulation, and control. When Cdk7 was deleted in mouse embryonic fibroblasts, cell cycle arrest occurred without any impact from CTD S5 phosphorylation, underscoring the importance of kinase activity for healthy cell division and growth. In the in vivo model, *Cdk* depletion led to embryonic mortality. In adults, the deficit results in the loss of stem cells in developing tissues and has no discernible impact on tissue that does not proliferate [121]. Despite being an important modulator of cell proliferation, CDK7 overexpression has been closely linked to various types of cancers [122,123,124]. Oncogenic TFs, often referred to as super enhancers, are responsible for developing many cancer types, and high transcription levels can accelerate their advancement [125,126].

### 5.1. Intrahepatic Cholangiocarcinoma (ICC)

Intrahepatic cholangiocarcinoma (ICC) is the second most common malignant liver tumor after hepatocellular carcinoma [127]. The most common treatment for ICC is surgery to remove the malignant tissue (curative resection); nevertheless, there is a significant risk of failure since the cancer cells may spread [128]. Because there are few viable therapeutic options, ICC has a high mortality rate and is extremely deadly. It has been investigated that ICC specimens exhibit overexpression of the kinase CDK7. CDK7 inhibition has been demonstrated to decrease the development of cancer cells and cause cell cycle arrest during the G2/M phase of the cell cycle. Additionally, there was a considerable decrease in the cancer cells’ ability to move and infiltrate. It has been demonstrated that the CDK7 inhibitor THZ1 inhibits the growth of ICC cells. In ICC cell lines, THZ1-mediated inhibition of CDK7 decreased cell proliferation, stopped the cell cycle, and prevented cancer cell migration and invasion [129].

### 5.2. Epithelial Ovarian Cancer

Over 90% of ovarian cancer is epithelial ovarian carcinoma (EOC), the most prevalent gynecological cancer [130,131]. Defects in cell cycle regulation are frequently seen in cancer cells, which promote unchecked cell growth and proliferation and ultimately lead to tumorigenesis. The kinase encourages the growth of tumors, and it has become a key target for the treatment of malignancies, including EOC [132,133]. The overexpression of CDK7 is directly proportional to the aggressiveness of EOC [134]. In EOC, CDK7 was inhibited using its small molecule inhibitor THZ1, which resulted in dysregulated gene transcription, inhibited E2F-regulated genes, and decreased EOC aggression [135].

### 5.3. Breast Cancer

Triple-negative breast cancer (TNBC) is a very complex, aggressive, and challenging type of breast cancer. The cells rely heavily on CDK7 and tCDKs. Given that TNBC has demonstrated susceptibility to CDK7 inhibitors, CDK7 is a key regulator and promoter of TNBC [136]. Breast cancer has extremely high expression of CDK-activating kinase (CAK), a triple complex made up of CDK7, MAT1, and Cyclin H develops [124]. High sensitivity to THZ1 was also seen in breast cancer that was HER2-responsive and HER2-resistant. To facilitate cell cycle progressions, CDK7 inhibits transcription, whereas HER2 controls the kinase’s transcriptional activity. In BC models resistant to HER2 inhibitors, dual inhibitors that target both HER2 and the kinase showed decreased tumor development [137]. CDK7 and ER mRNA levels were found to positively correlate in TCGA ER-positive (ER+) breast cancer samples, and patients with ER+ breast cancer who express high levels of CDK7 have a noticeably lower overall survival [138].

**Figure 5:**
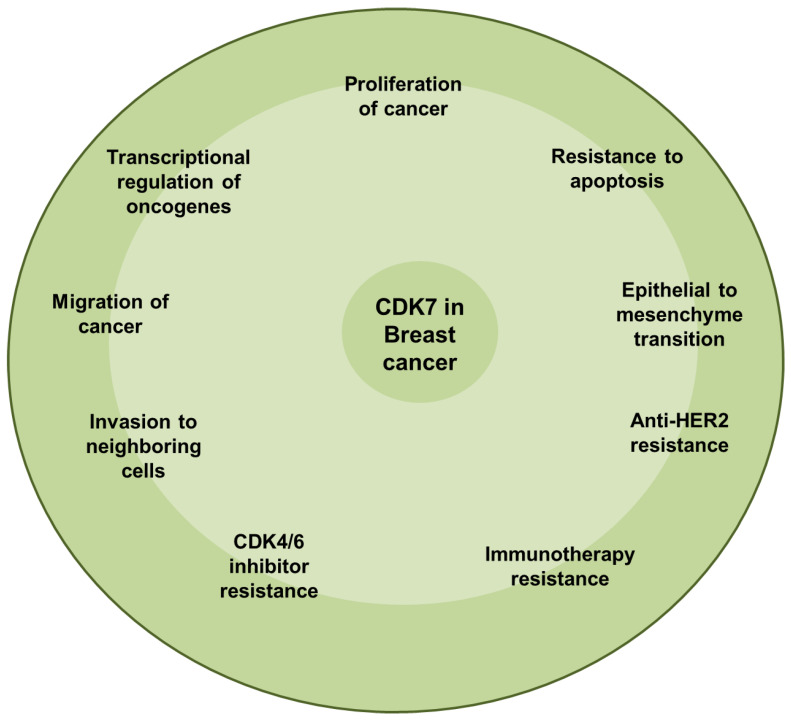
Role of CDK7 in breast cancer.

Treating TNBC by targeting CDK7 is a widely therapeutic option. Various small molecule inhibitors such as BS-181, THZ1, and others have been used successfully to suppress the TNBC malignancy. It is interesting to note that TNBC cells were more sensitive to CDK7 inhibition than HR+ breast cancer cells [139,140]. Figure 5 highlights the role of CDK7 in breast cancer.

### 5.4. Pancreatic Ductal Adenocarcinoma

More people die from pancreatic ductal adenocarcinoma (PDAC) than from any other solid tumor, making it a deadly cancer. CDK7 inhibition has emerged as a treatment approach for PDAC progression in several preclinical animals. Mechanistically, decreased gene transcription and cell cycle suppression are linked to kinase inhibition. One effective method for reducing the aggressiveness of cancer is to inhibit CDK7-dependent transcriptional addiction in the disease [141]. Prior to chemotherapy, kinase inhibition is linked to a decrease in DNA damage and an increase in cancer cell death. Mechanistically, blocking the kinase also prevents the activity of STAT3-MCL1-CHK1, which is linked to the survival and advancement of cancer [142,143]. Reduced MCL1 protein expression is linked to deletion of the CDK7 gene. THZ1 treatment in conjunction with chemotherapy showed a synergistic effect on decreased tumor survival rates. Inhibiting CDK7 also affects cell cycle regulation by lowering G2-M transition-related proteins such as WEE1 and other CDKs, which are likewise linked to decreased cell proliferation [144,145,146]. When WEE1 is inhibited, CDK1 is overexpressed, forcing cells to pass through G2-M checkpoints without properly repairing DNA damage [145].

## 6. Cyclin-Dependent Kinase 8 in Cancer

Through the phosphorylation of transcription factors and its kinase module, cyclin-dependent kinase 8 (CDK8) is essential for the control of transcription. The role of CDK8 in the initiation and spread of cancer has been demonstrated in numerous investigations. The CDK8 module, which consists of CDK8, CCNC, MED12, and MED13, is a 600 kDa complex in which CDK8 operates. This module forms unique “CDK8-Mediator” complexes that a reversible yet stable association with the mediator complex can biochemically purify [100,147,148]. The mediator–CDK8 module interacts via MED13 and specific, yet undefined, mediator subunits [149]. Depending on the cell type and situation, CDK8 can function as a tumor suppressor or an oncogene. Although its exact function is unknown, CDK8 helps keep cancers and embryonic stem cells in an undifferentiated state and stimulates cell proliferation via the serum response pathway [150,151].

### 6.1. Breast Cancer

A transcriptional co-regulator, CDK8, increases the activity of several TFs. The overexpression has been linked to the development of tumors and is frequently seen in breast cancer. Reduced relapse-free survival in patients with breast cancer is associated with elevated levels of CDK8 and its interacting proteins [149,152,153]. Research has shown that CDK8 expression and estrogen receptor (ER) levels in breast cancer are negatively correlated, suggesting that CDK8 may influence ER signaling pathways [154]. In ER-positive breast cancer cells, inhibiting CDK8 activity lessens estrogen’s transcriptional and growth-promoting effects. In order to promote the transcription of ER-regulated genes, CDK8 acts downstream of ER via increasing RNA Pol-II activity. CDK8 activity has been successfully reduced by tools like small-molecule inhibitors (like Senexin A and B) and genetic techniques like shRNA or CRISPR/Cas9, demonstrating its potential as a therapeutic target in breast cancer [155].

### 6.2. Colorectal Cancer β-catenin

CDK8 is a key promoter of tumor in colorectal cancer (CRC) by modulating the Wnt/β-catenin signaling pathway [156]. CDK8 is a kinase member of the mediator complex that acts as a transcriptional co-regulator, enhancing the transcription of oncogenic target genes, such as MYC and Cyclin D1. CDK8 is associated with up-regulating β-catenin activity, which aids in tumor development, invasion, and metastasis. CDK8 is a crucial player in the progression of CRC by up-regulating the transcription of *Wnt* target genes. Notably, CDK8 is a prospective therapeutic target in colorectal cancer, and inhibition of the kinase is associated with reduced tumor growth [157].

## 7. Cyclin-Dependent Kinase 9 as a Therapeutic Target in Cancer

Cyclin-dependent kinase 9 (CDK9) is the major regulator of the elongation phase of transcription. CDK9 is associated with various cancers linked to dysregulated transcriptional machinery. CDK9 aids the pause and release of RNA Pol II, which is a crucial and rate-limiting step in transcription. The step is found to be dysregulated in various types of cancers, and studies claim that CDK9 inhibition/degradation provides therapeutic benefits against cancer [158]. Overexpression of the kinase is associated with poor prognosis and confers resistance against chemotherapies. Some of the major types of cancers associated with CDK9 overexpression are myeloma, osteosarcoma, lung, breast, prostate cancer, and many more [106,159,160], which are explained here.

### 7.1. Pancreatic Cancer

Pancreatic cancer tissue shows overexpressed CDK9 and markedly reduced survival rates in patients. CDK9 expression is high in tumor tissue, particularly in well-differentiated malignancies [161]. The overexpression of the kinase is linked to a poor prognosis and is markedly overexpressed in pancreatic cancer tissues, especially in well-differentiated and chemoresistant subtypes. CDK9 promotes the survival of cancer cells by upregulating the expression of transient anti-apoptotic proteins, including Mcl-1 [162,163,164]. Selective inhibitors such as SNS-032 that inhibit CDK9 cause apoptosis and cell cycle arrest, upsetting the equilibrium between pro- and anti-apoptotic proteins. CDK9 inhibition also improves the effectiveness of traditional chemotherapies like gemcitabine and irinotecan, which may help overcome chemoresistance. CDK9 is a promising therapeutic target for the individualized therapy of pancreatic cancer, deserving of more preclinical and clinical research due to its ability to selectively target cancer cells and work in concert with conventional treatments [161].

### 7.2. Breast Cancer

Breast cancer frequently exhibits the dysregulation of CDK9, and its absence causes genomic instability [165]. The CDK9–cyclin T1 complex is essential for controlling RNA polymerase II’s transcriptional elongation, greatly increasing the transcriptional activity of important oncogenes like c-myc and myeloid cell leukemia 1 (Mcl-1). By encouraging metastasis through the maintenance of cancer stem cells (CSCs) and increasing epithelial–mesenchymal transition (EMT) processes, both of which are essential for tumor invasiveness and dissemination, these oncogenes propel the advancement of cancer [104,166,167]. Inhibition of CDK9-cyclin T1 has emerged as a new strategy against the therapeutic management of TNBC [168,169,170].

### 7.3. Osteosarcoma

The most prevalent primary malignant tumor of the bone is osteosarcoma. The prevalence of osteosarcoma is bimodal, increasing between the ages of 18 and 60, and it is marginally more common in men [171]. The high rates of patient recurrence and metastasis highlight the need for innovative therapeutic and preventive approaches to osteosarcoma, despite recent significant advancements in therapy. According to preclinical and clinical data, cyclin-dependent kinases (CDKs) regulate DNA transcription and cell cycle progression, which may be key factors in the initiation and spread of cancer [172]. Immunohistochemistry shows that a considerably shorter patient survival is linked to CDK9 overexpression. The most significant predictor of disease outcome for patients with osteosarcoma is the percentage of tumor necrosis following neoadjuvant chemotherapy, and it is inversely connected with CDK9 expression. In osteosarcoma, CDK9 knockdown with siRNA and CDK9 activity suppression using an inhibitor reduced cell growth and triggered apoptosis [159]. It has been noted that CDK9 overexpression and activation result in the production of oncogenes, such as MYC and MCL-1, which promote the growth and spread of sarcoma. It has been demonstrated that inhibiting CDK9 in sarcoma lowers the expression of these oncogenes as well as the development and proliferation of various sarcoma cells [173].

### 7.4. Ovarian Cancer

The most common cause of death for women with gynecological cancer diagnoses is ovarian cancer (OC). In general, it ranks as the fifth most common cause of mortality for women. Poor outcomes for this disease result from the majority of cases being detected at an advanced stage [174]. With a survival rate of only 30%, patients with ovarian cancer have a dismal outlook [175]. In an OC tissue microarray made with paired primary, metastatic, and recurrent tumor tissues from 26 OC patients, CDK9 expression was identified by immunohistochemistry. CDK9 was shown to be strongly expressed in human OC cell lines and raised in metastatic and recurrent ovarian tumor tissue. Furthermore, a negative patient prognosis was substantially connected with elevated CDK9. When CDK9 was inhibited by small interfering RNA (siRNA) or a CDK9 inhibitor, OC cell proliferation was decreased, apoptosis was promoted, and RNA transcription elongation was functionally hindered [176]. The tumor stage was higher in patients with higher CDK9 expression, suggesting that a higher CDK9 could predict the course of OC. The findings imply that CDK9 may be a promising therapeutic biomarker for ovarian cancer and is linked to the invasive development of human OC [177].

## 8. Cyclin-Dependent Kinase 12 in Cancer

CDK12 was initially identified by cDNA screens for cdc2 kinase-related cell cycle regulators. CDK12 is a tCDK that plays an important role in controlling the transcription of genes involved in cellular responses to stress and DNA damage, as opposed to CDKs, which facilitate the transition between various cell cycle stages. New research has shed light on CDK12’s possible clinical application as a biomarker and therapeutic target while also continuing to analyze its involvement in cell function and malignancy [91,178,179]. Oncogenes like *MYC* and *c-Fos,* as well as genes linked to super-enhancers (SEs), such as *RUNX1* and *GATA3*, are transcriptionally regulated by CDK12 [180,181,182]. *MYC* and *c-Fos* genomic alterations are frequently seen in malignancies and have been linked to the stimulation of carcinogenesis [183]. Numerous malignancies, including breast, ovarian, prostate, and gastric cancers, have been found to exhibit the aberrant expression or mutation of CDK12 [184,185,186]. Additionally, esophageal, endometrial, uterine, bladder, colorectal, and pancreatic ductal carcinomas are indirectly linked to CDK12 [91]. Interestingly, in various cancer types, CDK12 exhibits both tumorigenic and tumor-suppressive actions.

### 8.1. Breast Cancer

CDK12 expression in breast cancer is associated with enhanced tumorigenic properties and aggressive cancer phenotypes. Increased tumor growth, cell mobility, spheroid formation, and EMT are all associated with elevated CDK12 levels, and these factors all aid in the advancement of cancer. Moreover, a higher number of tumorigenic CD44+ cells in the CDK12 high group indicates that CDK12 promotes the preservation of breast cancer stemness and is connected to lung metastases. The positive correlation between CDK12 and c-myc expression emphasizes its function in triggering the c-myc/β-catenin signaling pathway, which may be a mechanism promoting the renewal of breast cancer stem cells [187]. CDK12 influences various signaling pathways, which in turn promotes the development of HER2-positive breast cancer [59]. By controlling cancer stem cells (CSCs) or influencing the genes required to activate downstream pathways such as ErbB-PI3K-AKT (Protein Kinase B) or WNT-signaling cascades, CDK12 facilitates cancer progression [188].

### 8.2. Ovarian Cancer

The MYC oncogene is amplified in over half of all tumors, one of the many copy number changes that define high-grade serous ovarian cancer. Since OC cells rely heavily on MYC to sustain their oncogenic development, MYC may be a promising therapeutic target for this challenging-to-treat cancer. By inhibiting CDK7, CDK12, and CDK13, MYC and OC are significantly reduced [189]. About 20% of high-grade ovarian tumors include mutations in the tumor suppressors BRCA1 and BRCA2, which encode proteins essential to homologous recombination (HR) repair. Nearly 50% of these cancers exhibit abnormalities in HR despite the fact that only 20% of them have mutations in BRCA1 or BRCA2. The catalytic activity of this kinase, which is involved in the transcription of a subset of genes, including BRCA1 and other DNA repair genes, is impaired by somatic CDK12 mutations seen in ovarian malignancies. Inhibiting CDK12 function in ovarian cancer cells lowers BRCA1 levels, interferes with HR repair, and makes the cells more susceptible to the drugs cisplatin and melphalan [190].

### 8.3. Prostate Cancer

The second most common cancer among men to be diagnosed is prostate cancer (PCa). Recently, it was shown that CDK12 is linked to PCa. It is believed that genomic instability caused by CDK12 deletion or mutation contributes to metastatic prostate cancer [191,192]. Furthermore, individuals with metastatic prostate cancer frequently have TP53, PTEN, and CDK12 abnormalities [193]. A whole-genome investigation of 101 metastatic PCa patients has demonstrated that CDK12, TP53, and BRCA2 inactivation impacts several types of structural variation in metastatic PCa. In particular, tandem duplications are linked to CDK12 mutations [194].

## 9. CDK 12/13 Inhibition as a Therapeutic Option in Cancer Treatment

Cyclin-dependent kinase 13 (CDK13) is implicated in transcriptional activation and has been proposed to phosphorylate RNA polymerase II. There is still much to learn about CDK13′s role in carcinogenesis and whether it catalyzes other protein substrates [195]. In a recent study, CDK12 and 13 inhibition was carried out, which led to suppressed expression of the DDR proteins and overall decreased breast cancer [111]. CDK13 is highly expressed in breast cancer tissues. Patients that expressed more CDK13 had larger tumors, distant metastases, and lymph node metastases at much greater rates. Patients with high expression levels of CDK13 have worse death and recurrence rates than those with low expression levels [196]. In ovarian cancer, the CDK12 and CDK13 genes are dysregulated, and their simultaneous up-regulation with the oncogene MYC indicates a poor prognosis. The remarkable sensitivity of ovarian cancer cells to CDK12/13 inhibition works in concert with clinically used cancer medications. Through defective splicing, dual CDK12/13 inhibition suppresses the expression of genes linked to cancer, according to transcriptome analysis. Inhibitors of the pathways controlled by these cancer-relevant genes (EGFR, RPTOR, and ATRIP) in conjunction with THZ531 therapy had a synergistic effect on cancer viability [197].

## 10. CDK 11

CDK 11 is a widely expressed nuclear enzyme and has roles in transcription, splicing, and modulations associated with cell cycle functions [85,86]. The overexpression of CDK11 is associated with tumor progression and poor survival of the patients. CDK11 inhibition or suppression studies show dependency on various cancers such as breast, osteosarcoma, ovarian, etc. CDK11 is also associated with enhancing the cytotoxicity of various anticancer drugs [114,198,199]. According to a recent study, OTS964 is the first CDK11 inhibitor discovered in medications with poorly defined targets that are in clinical and preclinical development. The OTS964 reduced the proliferation of multiple cancer cell lines, which is consistent with the findings of the CDK11 loss-of-function investigations. These findings support the idea that cancer cells rely on CDK11 and that the kinase may be targeted for cancer treatment [200]. Table 2 shows the association of CDK11 in various cancers.

## 11. CDK 19

In addition to binding to cyclin C, CDK19 is a paralog of CDK8. Additionally, CDK19 has kinase-independent functions in controlling the p53 stress response, and it can bind to the mediator complex to control transcription in a gene-specific way as well. More than 60 proteins phosphorylated by CDK8 and CDK19 were recently discovered by comprehensive proteomic research; many of these proteins are linked to transcription, DNA repair, and chromatin modification [76,207].

### Prostate Cancer

CDK19 has been strongly associated with prostate cancer due to its distinct and elevated expression in malignant and metastatic prostate tissues compared to normal tissues. A pan-cancer analysis using TCGA data revealed exceptionally high CDK19 expression in PC, correlating with disease progression and aggressive features, such as higher Gleason scores and advanced stages. Immunohistochemical analysis confirmed increased CDK19 protein levels in metastatic and castration-resistant prostate cancer (CRPC). CDK19 expression was also significantly associated with ERG rearrangements, an early event in prostate carcinogenesis, and correlated with Ki-67 expression, a marker of aggressive tumors. Functional studies demonstrated that CDK19 knockdown had limited effects on cell viability; it markedly reduced migration and invasion in prostate cancer cell lines, suggesting its role in metastatic progression. These findings highlight CDK19 as a potential therapeutic target in prostate cancer, particularly in advanced and metastatic cases, and underscore its importance in prostate cancer biology.

## 12. Conclusions

Cancer is a hard-to-treat disease caused by genetic, environmental, and a combination of both factors, making the treatment challenging. CDKs are a major protein kinase that is associated with regulatory roles in the cells; however, overexpression and mutations of these kinases are often associated with various types of cancers. CDKs play a diverse role in regulating cell functions, including cell cycle maintenance and transcription termed as cell cycle CDKs and transcriptional CDKs. Through their ability to phosphorylate the RNA polymerase II C-terminal domain (CTD), transcription-associated CDKs have become crucial players in cancer biology. Significant attention has been paid to the role that transcriptional CDKs play in cancer, including CDK7, CDK8, CDK9, CDK12, and CDK13. These kinases affect gene expression profiles that are vital for cell survival and proliferation by controlling vital transcriptional events such as initiation, elongation, and termination. In conclusion, the knowledge of transcription-associated CDKs is a fascinating and promising field in oncology that will probably continue to receive a lot of interest in the years to come. We look forward to the development of new synthetic lethal techniques to target tumors characterized by the loss of these crucial regulators, as well as the introduction of transcription-associated CDK inhibitors into the clinical arsenal against cancer.

## Figures and Tables

**Table 1:** Transcriptional CDKs and their role in cancer.

CDK Name	Functions	Expression in Cancer	References
CDK7	- Phosphorylates Ser5 of RNAP II CTD to start transcription. - Participates in the repair of DNA. - As a component of the CAK complex, it controls the cell cycle.	- Highly expressed in lung, ovarian, and breast malignancies. - Therapy resistance and tumor development are associated with elevated CDK7 levels.	[28,97,98,99]
CDK8	- Utilizes the mediator complex to regulate transcription. - Functions as a repressor or co-activator of transcription. - Wnt/β-catenin signaling is modulated.	- Overexpressed in melanoma, breast, and colorectal cancer. Uses oncogene regulation to encourage tumorigenesis and metastasis.	[100,101,102,103]
CDK9	- As a component of the P-TEFb complex, it phosphorylates Ser2 of RNAP II CTD, facilitating transcription elongation and regulating genes involved in the stress response.	- Overexpressed in malignancies of the breast, prostate, and leukemia. - Promotes the survival and growth of cancer cells.	[104,105,106,107]
CDK12	- Modulates the transcription of genes that respond to DNA damage. - Phosphorylates RNAP II CTD’s Ser2. - Preserves the stability of the genome.	- Mutated in prostate and ovarian malignancies; loss of function results in genomic instability and poor homologous recombination repair.	[108,109,110]
CDK13	- Phosphorylates Ser2 of RNAP II CTD. - Splicing and transcription of complex genes. - Regulates mRNA processing.	- Overexpression and mutations in colorectal, endometrial, and breast malignancies linked to splicing and transcriptional errors that are implicated in carcinogenesis.	[111,112,113]
CDK11	- Associated with transcription, RNA processing, and splicing. - Exists as isoforms (CDK11p58 and CDK11p110). - Regulates mitosis.	- Overexpression of CDK11p110 is seen in prostate and breast malignancies. - CDK11p58 stimulates mitotic defects and apoptosis in cancer cells.	[114,115,116]
CDK19	- A paralog of CDK8. - Controls mediator-dependent transcription; has a role in the transcription of particular genes and the stress response.	- Elevated in colorectal and breast malignancies. In contrast to CDK8, it might control different transcriptional programs in cancer.	[117,118,119]

**Table 2:** CDK11 in various cancer types.

Cancer Type	Expression and Association	Effects of CDK11 Knockdown	Therapeutic Implications	References
Breast Cancer (BC)	Overexpressed in BC cell lines and tumor tissues; associated with poor prognosis, advanced TNM stage, and poor differentiation. There is 100% staining and high expression in TNBC in patient tissues.	Reduces viability and clonal survival; causes apoptosis; and inhibits growth and migration. Tumors are reduced by in vivo treatment.	Promising target for BC, particularly TNBC.	[201,202]
Multiple Myeloma (MM)	Relative to normal tissues, overexpressed in initial myeloma tissues. Determined using RNAi lethality screening to be a survival gene.	Its function as a survival gene is supported by inhibition, which lowers viability.	Represents a novel therapeutic strategy for myeloma.	[203,204]
Osteosarcoma	High expression in osteosarcoma cells is linked to a lower survival rate for patients.	Causes apoptosis and cell death; inhibits growth, migration, and invasion. The findings of CRISPR-Cas9 silencing are comparable.	The development of CDK11 inhibitors may help treat osteosarcoma.	[205,206]
Liposarcoma	Compared with benign lipomas, overexpressed in liposarcoma tissues.	Increases doxorubicin cytotoxicity while decreasing proliferation and inducing apoptosis.	Additional pathway research is necessary to determine whether CDK11 is a viable therapeutic target.	[199]
